# Commentary on Autism and the double‐empathy problem: Implications for development and mental health

**DOI:** 10.1111/bjdp.12410

**Published:** 2022-03-26

**Authors:** Beatriz López

**Affiliations:** ^1^ Department of Psychology University of Portsmouth Portsmouth UK

Mitchell et al.’s proposal that poor mental health in autism may stem from recurrent poor social interactions with non‐autistic people across the lifespan, due to what Milton (2012) has termed as the double‐empathy problem, is both timely and welcomed. It adds to the increasing voices advocating for a reconceptualization of autism as a condition that ‘*is both biologically and socially derived*’ (Milton, 2012, p. 866). Specifically, the authors question the validity of focusing the level of study of autism exclusively on the autistic person. In this respect, the authors stress the need to investigate, first, social interactions between autistic and non‐autistic populations and, second, their impact over time on the mental health of autistic people.

The calls for exploring the nature of social interactions between autistic and non‐autistic people are gaining momentum, and the authors make a powerful argument for the need to do so. Specifically, they delineate a well‐reasoned timeline that leads from being poorly understood by non‐autistic people to feelings of loneliness and the lack of a sense of belonging, which in turn, result in poor mental health. Although this proposal is speculative in nature, it has the potential of having a significant impact on our understanding of autism as a condition that develops within, and is shaped by, its intersection with a non‐autistic environment (López, 2015). That is, reframing autistic people's challenges as emerging at the intersection of autistic characteristics and a non‐autistic society.

Most importantly, the proposal has the potential to broaden our understanding of the factors at play in social interactions, both in autism and typical development. So far research on social interactions has focused primarily on the cognitive process associated with the understanding of social situations and information, that is, on social cognition. Social cognition overwhelmingly favour the notion that, as minds are opaque, we understand others by inferring their inner mental states, what has been termed Theory of Mind ability (ToM; Premack & Woodruff, 1978). As the ToM approach does not place special importance on the role of social interactions in the processing of social information, it employs methods in which participants, are not placed in *actual* social interactions, such as theory of mind tasks (Baron‐Cohen et al., 1985), retrodictive inference tasks (Gallese & Goldman, 1998) or eye‐tracking studies using pictures/films of social scenes (Klin et al., 2002). Through the use of these methods we have gained invaluable knowledge regarding how, as mere observers, we process social information. More relevant to autism, we have learned that autistic people have difficulties in ‘reading the minds’ of non‐autistic people, and vice versa, that non‐autistic people have difficulties interpreting autistic people (Sheppard et al., 2016).

However, the ability to cognitively process social information (i.e., ToM), is only one factor contributing to our understanding of others. As the authors rightly mention, an alternative theoretical approach, the second‐person approach, proposes that social interactions themselves are key to our ability to understand others (Di Paolo & Jaegher, 2017; Hobson, 2004; Reddy, 2009; Trevarthen, 1979; Zahavi, 2001). This approach argues that the kind of implicit understanding we gain from others by interacting with them is qualitatively very different from understanding others as mere observers. Although this distinction may seem a trivial matter a quick look at the literature proves otherwise. For instance, eye‐tracking studies where participants are shown social scenes consistently show a bias towards faces (Fletcher‐Watson et al., 2009). In contrast, in the presence of social partners we actively avoid looking at people (see Risko et al., 2012 for a review). Merely telling participants that they are watching real people through a live webcam, as opposed to telling them they are watching a video, is enough to remove the preference towards looking at faces (Gregory et al., 2015). Surprisingly, and as an aside, despite the consistent evidence that the mere presence of social partners influences behaviour it is still common to read articles indistinctively discussing research evidence without making an explicit distinction as to whether it comes from studies where social partners are present or not.

Although these studies are a step forward, they still fall short of providing a full understanding of the nature of social interactions. In particular, these studies still focus the level of study on participants’ individual performance, and not on the social interaction itself. Hence the importance of the authors’ proposal to study the dynamics of social interactions between autistic and non‐autistic people. At the end of the day, what research ultimately aims to explain is the difficulties autistic people face when interacting with non‐autistic people and how these difficulties can be ameliorated. More importantly, the authors stress the need to understand these difficulties from a developmental perspective. Autism is by definition a developmental condition, which evolves over the lifespan as a result of genetic factors in interaction with the environment. Given that the core deficit in autism relates to social communication and interaction, it makes sense, as the authors state, to focus research specifically on how this deficit influences their social interactions with non‐autistic partners from an early age. Due to the dominance of ToM approaches over the last few decades few studies have examined social interactions between autistic children and their caregivers early in life. This research points to reduced periods of engagement in the first 6 months of life as a result of infants producing fewer vocalizations (Apicella et al., 2013). A matter of concern if we consider that reduced opportunities to engage in social interactions would significantly aggravate existing innate social deficits. On a more positive note, research also provides evidence of the ingenious ways in which parents effectively adapt their communication to the needs of their children (Doussard‐Roosevelt et al., 2003; Kasari et al., 1988). Unfortunately, studies examining caregivers and autistic infants’ dyads, or autistic–autistic and autistic–non‐autistic dyads are rare despite their potential to identify optimal patterns of communication, to avoid later in life, potential mental health difficulties. It is primarily for this reason that this article is of particular significance.
